# Diagnostics and Therapeutics in Early Stage Breast Cancer Receiving Neoadjuvant Systemic Therapy

**DOI:** 10.3390/cancers15194874

**Published:** 2023-10-07

**Authors:** Paolo Belli, Simone Palma, Melania Costantini

**Affiliations:** Department of Bioimaging, Radiation Oncology and Hematology, UOC of Radiologia, Fondazione Policlinico Universitario A. Gemelli IRCSS, Largo A. Gemelli 8, 00168 Rome, Italy; simone.palma@guest.policlinicogemelli.it (S.P.); melania.costantini@policlinicogemelli.it (M.C.)

Breast cancer (BC) remains a major challenge for oncology today, impacting the lives of countless individuals worldwide. Yet, in the journey toward improved outcomes and personalized care, a beacon of hope has emerged in the form of neoadjuvant systemic therapy (NST). NST is used in early stage BC to predict outcomes and increase eligibility for breast conserving surgery, recommended for patients with triple negative (TN) or human epidermal growth receptor 2 positive (HER2+) diseases. Magnetic resonance imaging (MRI) is an essential tool for assessing breast tumors, axillary lymph node status and response to NST, aiding in surgical decision-making. The goal of this Special Issue is to analyze closely related cutting-edge diagnostic and therapeutic approaches in early stage BC receiving NST, based on a collection of review and research articles with the latest evidence.

As we said, neoadjuvant systemic therapy has revolutionized the approach to early stage BC in HER2+ and TN patients. Giffoni et al. [1] provided a comprehensive overview of the evolving landscape of systemic treatment approaches for early stage HER2+ BC. The review synthesizes current research findings and insights from the literature, highlighting the latest evidence around early HER2+ BC. They point out that categorizing patients into BC risk groups, evaluating the tumor and lymph node (LN) staging, is crucial in choosing the correct therapeutic approach. In patients with low-risk disease with tumors measuring up to 20 mm and LN negative, NST can be avoided, while in the high-risk category comprising larger tumors or LN positive, NST would be useful to downstaging disease.

TN BC is known for its aggressive nature, poor prognosis, and early relapse and historically treatment has heavily relied on chemotherapy. However, recent advancements in biologic and targeted therapies are reshaping early stage TN BC treatment strategies as showed by Garufi et al. [2]. The authors gathered and enhanced recent evidence suggesting that the addition of carboplatin to anthracycline/taxane NST shows promising results, including improved pathological complete response (pCR) rates and long-term outcomes. Also, immunotherapy, particularly with immune checkpoint inhibitors, is proving significant improvements in pCR rates and long-term outcomes. Given the costs and potential immune-mediated toxicities associated with immunotherapy, patient selection is critical and subgroup analyses suggest that patients with more advanced disease stages benefit the most from the addition of immune checkpoint inhibitors to NST.

In their research article, Rapoport et al. [3] highlighted the importance of Tumor-Infiltrating Lymphocytes (TILs) as an indicator of the immune response within the tumor microenvironment in early breast cancer. They evaluated Immunoscore^®^, a classification system that incorporates the number, type, and distribution of CD3+ and CD8+ immune cells. The score was initially developed for the prognosis of colon cancer, but it is now widely used in clinical research for prognostic and predictive evaluation in many types of solid tumors. As a first clinical validation of the prognostic potential of the Immunoscore^®^ in patients with BC, the authors assessed this test in BC in the neoadjuvant setting. The presence of high CD3+, CD8+ TILs, and a high Immunoscore^®^ is predictive of better treatment responses. Patients with these characteristics are more likely to achieve pCR, which is a favorable prognostic index. These findings have significant clinical implications, as they suggest that assessing TILs and Immunoscore^®^ could help tailor treatment strategies for BC patients.

MRI is an essential diagnostic tool for evaluating NST response and can influence the surgical management. In their reviews, Panico et al. and Conti et al. [4,5] emphasize that accurate staging with MRI leads to better treatment strategies and improves patient care, ultimately impacting patient outcomes positively. They underscore the importance of precision in surgical decisions and the need for a personalized approach to optimize patient care and outcomes by analyzing the different surgical approaches, discussing the role of axillary surgery, as well as the possibility of non-operative management after-NST, which has been the subject of recent trials. Finally, they gathered recent works about emerging techniques that will soon change the diagnostic assessment of breast cancer such as ultrafast Breast MRI, Contrast-Enhancement Mammography, Radiomics, and Machine Learning.

The use of MRI can also be useful for assessing the response to NST in specific biological tumor subtypes as demonstrated in the research article of Panthi et al. [6]. They assessed Functional Tumor Volumes (FTVs) derived from Dynamic Contrast-Enhanced MRI (DCE-MRI), that provide a dynamic and detailed view of tumor vascularization and response to treatment, as predictors of response in TNBC. FTV measurements from DCE MRI are useful biomarkers for discriminating TNBC patients with pCR and non-pCR to NST. The study showed that FTVs by DCE MRI after two cycles of treatment can predict the treatment response with high performance like that of FTVs after four cycles of treatment, thus providing an earlier opportunity for modifying the patient’s treatment course with early identification of non-responders.

In parallel with the necessity of an adequate “T” staging after NST, it is also important to define the “N” stage, indeed it represents the main prognostic factor affecting the rate of recurrence and the therapeutic management so that a correct staging of the axillary lymph node status is fundamental. Di Paola et al. [7] in their review article analyze the crucial role of ultrasound (US) and MRI, considering the high false negative rate of clinical examination (up to 45%). According to the authors, current evidence agrees that ultrasound is currently considered the gold standard for the assessment of the axillary cavity, due to its high spatial resolution and high negative predictive value; indeed, less than two nodes involved, in T1 and T2 tumor, will address to sentinel lymph node biopsy (SLNB) instead of axillary lymph node dissection (ALND). The role of MRI is also being highlighted, as although it has a lower spatial resolution, it provides an overview of the ‘T’, and of contralateral lymph node status. In another review work, Di Paola et al. [8] gave an overview of image-guide techniques for localizing metastatic lymph nodes at diagnosis in patients who will be treated with NST. In the NST setting marking positive lymph nodes (LNs) at the time of diagnosis allows for their removal during post-NST surgery because the LNs at diagnosis do not always coincide with SLNs after NST. For this reason, the targeted axillary dissection (TAD), an axillary staging technique that combines the removal of the metastatic LNs clipped before NST and SLNB, has developed reducing the false negative rate of SLNB alone. The knowledge of different types of localization methods and their relative advantages and disadvantages is crucial to orientate the choice among the most appropriate method. The authors finally suggest that the development of more integrated international guidelines on the choice of individual localization methods and their clinical indications seems appropriate.

In a prospective research article, Rella et al. [9] explore the accuracy of the Radio-Guided Occult Lesion Localization (ROLL) technique for biopsy-proven metastatic axillary LNs staging after NST, applied on positive LNs marked with a clip before the beginning of NST. The authors found that the ROLL procedure for clipped LNs can be proposed for axillary nodal staging after NST in patients with node-positive BC at diagnosis, with a false negative rate of 3.13%.

Finally, an important clinical issue to consider in selecting NST in BC is the likelihood of cancer recurrence. Rabinovici-Cohen et al. [10], in their retrospective study of a large cohort of patients, assessed the prediction of cancer recurrence in patients with locally advanced BC treated with NST, using a multimodal prediction model based on clinical data and multiparametric MRI images before NST. The authors found that the multimodal model offers improved results over the unimodal models and used interpretability methods to explain the model and identify important clinical features for predicting recurrence such as body mass index, age at diagnosis, and HER2 expression.

The combination of these research and review articles collected in this Special Issue give an overview that allows us to better comprehend the wide and heterogeneous field of neoadjuvant treatment in early stage breast cancer. They provide a detailed and up-to-date view of the main advances in the field of pharmacological neoadjuvant treatment in early stage BC, and also in relation to tumor molecular subtypes, tumor staging (T) and lymph node assessment (N).

The possibility of predicting pathological complete response and recurrence rate using clinical and radiologic information was also discussed promising new frontiers in personalized care and outcome.
